# Quantitative analysis of colon-lengthening techniques in laparoscopic low rectal cancer surgery with left colic artery preservation: a fresh cadaveric model study

**DOI:** 10.1007/s00595-026-03354-5

**Published:** 2026-06-04

**Authors:** Yunjie Shi, Jing Wu, Minxin Gao, Bing Liu, Hao Wang

**Affiliations:** Department of Colorectal Surgery, The First Affiliated Hospital (Changhai Hospital), Naval Medical University, Shanghai, 200433 China

**Keywords:** Left colic artery preservation, Bowel lengthening, Splenic flexure mobilization, Inferior mesenteric vein ligation, Tension-free anastomosis, Low rectal cancer

## Abstract

**Purpose:**

To quantify colonic lengthening achieved by complete splenic flexure mobilization (SFM) and high inferior mesenteric vein (IMV) ligation while preserving the left colic artery (LCA) in laparoscopic low rectal cancer surgery.

**Methods:**

Standardized LCA-preserving anterior resection was performed on 18 fresh cadavers. The additional colonic length gained after each maneuver was measured under tension-free conditions. High inferior mesenteric artery (IMA) ligation, which sacrifices the LCA, was also measured as a reference comparator.

**Results:**

The mean baseline resection length was 10.4 ± 6.9 cm. Additional gains: 6.3 ± 1.7 cm after SFM and 14.4 ± 2.1 cm after high IMV ligation (maximum gain under LCA preservation). As a reference, high IMA ligation (without LCA preservation) provided a lengthening of 18.4 ± 1.7 cm. In malrotation (*n* = 2), IMV ligation yielded only 10.2–12.3 cm of mesenteric length. BMI correlated positively with the baseline length (*r* = 0.529, *p* = 0.023).

**Conclusions:**

Under LCA preservation, high IMV ligation provides the greatest lengthening (14.4 cm), although efficacy is reduced with malrotation. These data guide preoperative planning for tension-free anastomosis and adequate margin placement.

## Introduction

During laparoscopic low rectal cancer surgery, the surgical approach of preserving the left colic artery (LCA) during No. 253 lymph node dissection has gained increasing acceptance among scholars. Studies have indicated that preserving the LCA can improve blood supply to the anastomotic site, thereby reducing the risk of anastomotic leakage. This is particularly significant for patients who have undergone neoadjuvant chemoradiotherapy, as the treatment causes microvascular damage to the rectal mucosa and reduces the intestinal blood supply, impairing anastomotic healing. Therefore, preserving the LCA holds enhanced clinical value for this patient population [1].

During laparoscopic surgery for low rectal cancer, meticulous planning of the mobilization length of the colon and the extent of mesenteric trimming is required in advance. This ensures that while preserving the LCA, we can achieve both tension-free anastomosis and adequate proximal and distal rectal resection margins. A study revealed that in low rectal cancer resection and anastomosis procedures, the incidence of anastomotic leakage was 31.3% in the tight bridging group, 20% in the sagging bridging group, and 2.2% in the resting group (*P* = 0.002) [2]. This underscores the imperative of pursuing truly tension-free anastomoses to ensure anastomotic integrity. Moreover, ensuring adequate colon resection margins is fundamental to achieving a surgical radical cure, with the proximal margin length serving as a critical quality indicator for rectal cancer resection surgery. Morikawa et al. demonstrated that in low rectal cancer, 29.2% of proximal positive lymph nodes were located > 5 cm superior to the tumor’s upper border, while 1.5% extended beyond 10 cm [3]. The author advocates a proximal resection margin of approximately 10 cm as the optimal therapeutic strategy.

Splenic flexure mobilization (SFM), which involves division of the phrenocolic, splenocolic, and gastrocolic ligaments, is essential to release the colon’s most fixed point, enabling tension-free anastomosis in left-sided resections [4]. High ligation of the inferior mesenteric artery (IMA) at its aortic origin facilitates medial-to-lateral dissection, whereas division of the inferior mesenteric vein (IMV) near the pancreatic border further mobilizes the mesenteric root [5]. Together, these steps synergize to optimize colonic reach and minimize the risk of anastomosis. Given the associated surgical risks of these maneuvers to gain colonic length, particularly with LCA preservation, surgeons must preoperatively estimate both the total required colonic length and the length contributed by each maneuver to ensure tension-free anastomosis and adequate surgical margins. The correlation between the additional colonic length achieved through these maneuvers and the length required for LCA-preserving laparoscopic low colorectal anastomosis has not yet been well characterized.

In this study, we performed laparoscopic transabdominal low rectal resection simulations in fresh human cadavers to investigate the bowel resection length, quantify the additional length gained through various colonic lengthening maneuvers, and evaluate their combined effectswhile preserving the LCAto achieve a completely tension-free anastomosis and adequate surgical margins.

## Materials and methods

### Fresh tissue cadaver

The study protocol was approved by the regulatory board of the Fresh Tissue Cadaver Laboratories, Naval Medical University, China. All cadavers were fresh, maintained at approximately 1 °C, and underwent only minimal formalin fixation. Fresh human cadavers were brought from cold storage 6 h before the laboratory session to allow time to reach room temperature. High-definition laparoscopy was used for dissection of all cadavers.

### Surgical technique

**Fig. 1:**
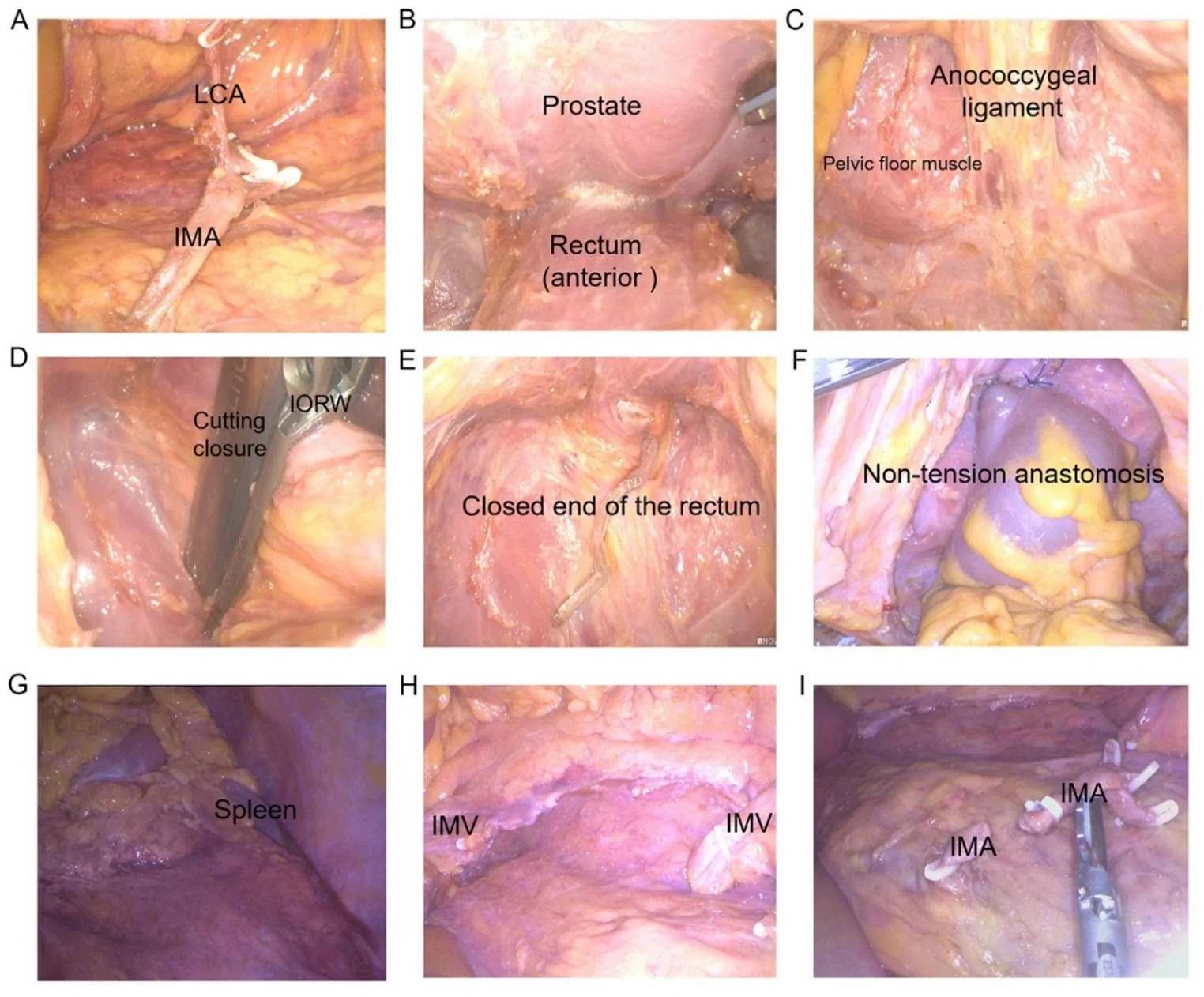
Laparoscopic intestinal lengthening protocol with LCA preservation for rectal resection and anastomosis. (**A**) Preservation of LCA; (**B**) Dissection of the anterior wall of the rectum; (**C**) Dissection of the posterior wall of the rectum; (**D**) IORW and cut closured of the rectum; (**E**) The residual end of the rectum at the pelvic floor; (**F**) Non-tension anastomosis; (**G**) Complete SFM; (**H**) High ligation of the IMV; (**I**) Ligation at the origin of the IMA

Initially, standardized laparoscopic transabdominal anterior resection sequences were performed [6]. We performed standardized D3 lymph node dissection for No. 253 according to the protocol, with boundaries defined as follows: cephalad border at the level superior to the origin of the IMA, medial border along the main trunk between the IMA and LCA origins, lateral border at the medial edge of the IMV, and caudal border extending from the LCA origin to its crossing point with the IMV. The dissection proceeded distally along the IMA root until the LCA origin was exposed. The IMA was then ligated and divided at a low position (distal to the LCA origin), followed by complete skeletonization along the course of the LCA and exposure of the IMV, ensuring thorough clearance of the No. 253 lymph nodes (Fig. 1A). Anterior rectal dissection was initiated by incising the lowest point of the peritoneal reflection, preserving Denonvilliers’ fascia integrity, and extending to expose either the prostate or vagina (Fig. 1B). Posterior rectal dissection was performed to the pelvic floor muscles, with subsequent exposure of the anococcygeal ligament (Fig. 1C). Following complete rectal mobilization, the rectal lumen was occluded and transected at the level of the pelvic floor, immediately above the anorectal ring, to simulate the distal resection margin for low rectal cancer. Intraoperative rectal washout (IORW) was performed, after which the distal rectum was cut and closed (Fig. 1D, E). IORW has long constituted an essential step in sphincter-preserving surgery for rectal cancer and remains a critical component of aseptic techniques and oncological principles in radical rectal cancer resection [7]. This standardized distal transection level, defined by the pelvic floor musculature, served as a fixed anatomical reference point for all subsequent length measurements. We mandated completely tension-free anastomosis as critical for preventing anastomotic leakage (Fig. 1F). Subsequently, we performed complete SFM (Fig. 1G), high ligation of the IMV(Fig. 1H), and ligation at the origin of the IMA (Fig. 1I) to quantify the resultant colonic lengthening.

### Measurement method

**Fig. 2:**
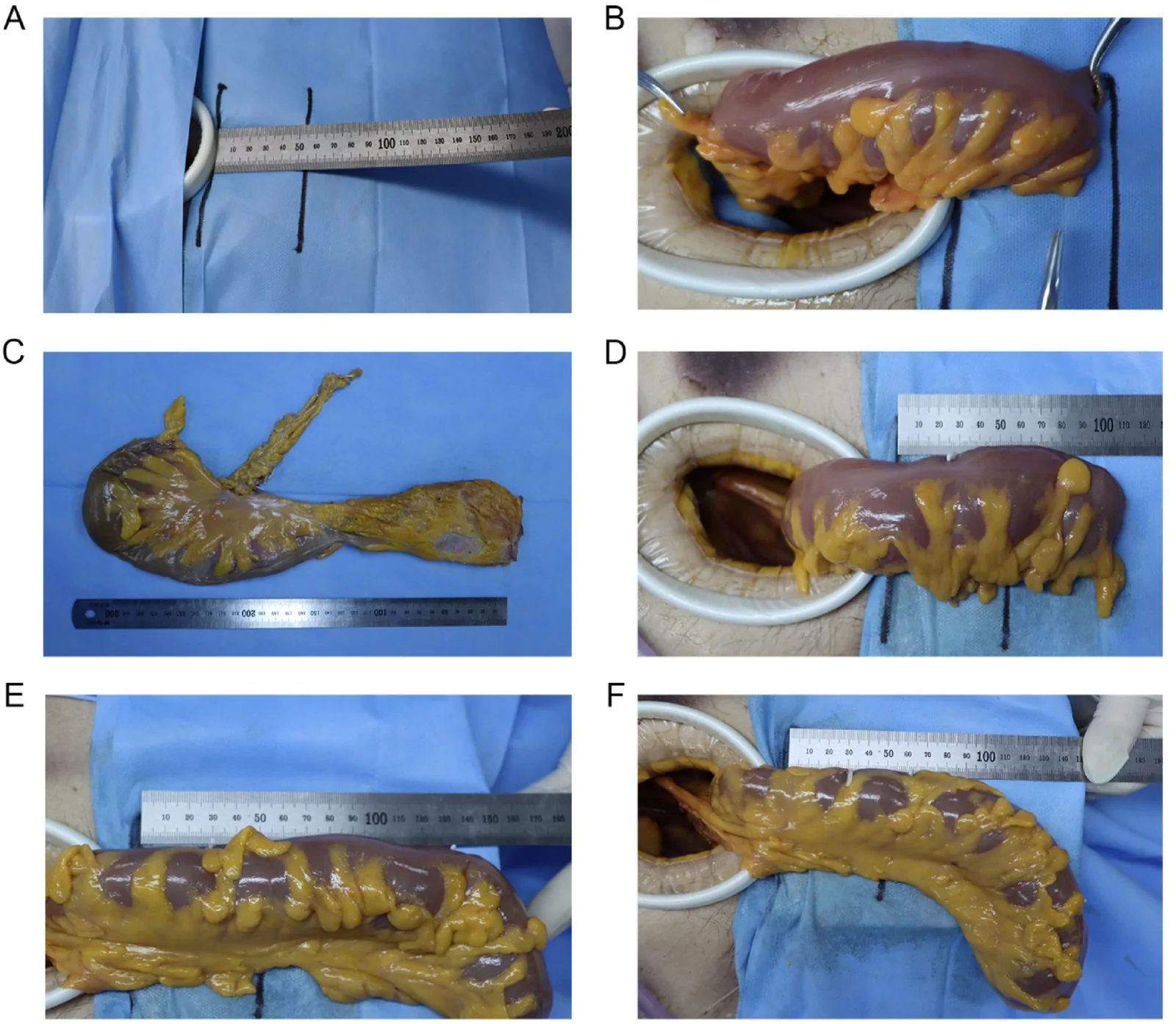
Methods for quantifying bowel lengthening. **A**. The superior and inferior borders of the pubic symphysis were marked on the skin; **B**. Localizing the proximal transection level upon achieving absolute tension-free anastomosis; **C**. Resected bowel segment achieving complete tension-free anastomosis with preserved LCA; **D**-**F**. Lengthened colonic segment achieved through complete SFM, high ligation of the IMV, and ligation of the IMA at its origin

Following the completion of laparoscopic manipulation, the superior and inferior borders of the pubic symphysis were marked on the skin (Fig. 2A). The intestinal tract was exteriorized through the incision, and the intended anastomosed intestinal segment was gently pulled down toward the pelvis. The condition of ‘absolute tension-free anastomosis’ was defined by two objective criteria: [1] the proximal bowel could be advanced to the level of the inferior border of the pubic symphysis (a fixed anatomical landmark) without any visible stretching or blanching of the mesentery or bowel wall, and [2] no additional traction force was required to maintain this position. Under these standardized conditions, the proximal bowel was transected precisely at the point where it first reached the inferior border of the pubic symphysis (Fig. 2B, C).

Subsequently, we performed complete mobilization of the splenic flexure, high ligation of the inferior mesenteric vein (IMV), and ligation of the inferior mesenteric artery (IMA) at its origin. Following each maneuver, a Hem-o-lock clip was applied to the lengthened colon segment to quantify the resultant colonic lengthening (Fig. 2D-F).

### Statistical analysis

Data were evaluated for correlation using Pearson and Spearman correlation coefficients. If both datasets possessed a normal distribution, the Pearson correlation coefficient was used. If one of the datasets was normally distributed, the Spearman correlation coefficient was used. Statistical significance was set at *p* < 0.05.

## Results

Initially, we analyzed the resected bowel length, proximal/distal margins, and lymph node yield from laparoscopic rectal cancer surgeries performed by our team with preserved LCA. The results demonstrated that intraoperative decision-making based on bowel length and mesenteric width determined SFM and/or high ligation of the IMV/IMA, ensuring adequate resection margins and lymphadenectomy (Table 1).

**Table 1:** Clinical resection length of rectal specimen

	LCA Preserved, *n*=85	Splenic flexure Mobilization, *n*=50	High IMA Ligation, *n*=60
Length of resection, cm	15.42 ± 3.59	15.38 ± 3.61	16.55 ± 3.56
Proximal margin, cm	9.92 ± 3.42	9.91 ± 3.42	9.40 ± 3.37
Distal margin, cm	2.83 ± 1.39	2.82 ± 1.41	3.69 ± 1.73
Lymph nodes dissected, amounts	14.51 ± 4.75	14.62 ± 4.78	17.25 ± 5.61

This study included 18 fresh cadavers. Among them, 14 were men and four were women. Based on post-mortem measurements, the mean height and weight were 169.4 ± 8.5 cm and 66.9 ± 9.8 kg, respectively, with an average body mass index (BMI) of 23.2 ± 2.2 kg/m².

The mean length of the resected bowel was 10.4 ± 6.9 cm while preserving the LCA and achieving complete tension-free anastomosis. When splenic flexure takedown was added to LCA preservation, an additional length of 6.3 ± 1.7 cm was gained. When IMV ligation at the level of the ligament of Treitz and mobilization of the transverse mesocolon off the inferior border of the pancreas were added, an additional 14.4 ± 2.1 cm of length was gained compared to LCA preservation. In cases ID 4 and ID 5, significant malrotation of the left colon was observed, with a notably narrow left colonic mesentery. High ligation of the IMV resulted in colonic lengthening of 12.3 cm and 10.2 cm. Finally, high ligation and transection of the IMA at its root, compared with preservation of the LCA, resulted in an intestinal lengthening of 18.4 ± 1.7 cm (Table 2).

When the correlations between height, weight, BMI, and resection length were evaluated, BMI showed a positive correlation with resection length (correlation coefficient = 0.529, *P* = 0.023). However, no significant correlations were found between the height, weight, BMI, and resection length achieved using SFM, high IMV ligation, or high IMA ligation (Table 3).

**Table 2:** Length gained after lengthening procedures

Cadaver	Length of resection, cm	Length gained, cm		
ID	LCA preserved	Splenic flexure mobilization	High IMV ligation	High IMA ligation
1	5.2	8.9	16	19.1
2	17	6.6	14.9	17.2
3	11.7	5.6	14.8	18.3
4	9.6	7.2	12.3	18.7
5	12.9	4.3	10.2	15.9
6	10.4	4.2	11.7	14.0
7	9	4.5	14.3	18.3
8	10.8	9.4	16.4	19.2
9	10.7	6.2	16.6	20.4
10	5.3	5.4	14.2	17
11	8.8	6.8	16.6	19.7
12	6.7	9.6	16.9	20.4
13	5.1	5.2	12.2	16.1
14	5.6	5.0	13.2	17.2
15	10.3	7.3	15.3	18.5
16	4.9	3.5	11.5	16.8
17	8.9	6.8	15.3	19.1
18	35	7.5	17.5	20.5
Mean±SD	10.4±6.9	6.3±1.7	14.4±2.1	18.4±1.7

**Table 3:** Correlation between mobilization procedures and anthropomorphic measurements

Measurements	Length of resection	Splenic flexure mobilization	High IMV ligation	High IMA ligation
Weight(kg)	CC 0.322, ^a^ *P*=0.193	CC -0.171, ^b^ *P*=0.498	CC 0.028, ^b^ *P*=0.911	CC -0.251, ^b^ *P*=0.510
Height(cm)	CC-0.035, ^a^ *P*=0.889	CC -0.218, ^b^ *P*=0.384	CC -0.239, ^b^ *P*=0.339	CC-0.369, ^b^ *P*=0.132
BMI	**CC 0.529**, ^**a**^ *P*=0.023	CC -0.065, ^b^ *P*=0.798	CC 0.312, ^b^ *P*=0.208	CC -0.020, ^b^ *P*=0.936

^a^ Spearman correlation coefficient

^b^ Pearson correlation coefficient

## Discussion

During rectal cancer surgery, the ligation of the IMA can be divided into two approaches: one involves high ligation at the origin of the IMA (HT), first proposed by Miles in 1908 [8]; the second is low ligation distal to the origin of the LCA (LT), a technique preserving the LCA that has gained increasing acceptance among scholars in recent years [9, 10]. The significance of preserving the LCA in rectal cancer surgery lies in: (1) improving anastomotic blood supply to reduce the risk of anastomotic leakage [11]; (2) protecting the LCA bifurcation is crucial for patients with compromised blood flow at the splenic flexure watershed area [12]; (3) technically, preserving the LCA during rectal cancer surgery does not compromise the dissection of No. 253 lymph nodes [13]. Mesenteric anastomotic tension is a critical factor affecting anastomotic healing. In clinical practice, high ligation of the IMA facilitates obtaining sufficient colonic length for tension-free anastomosis [14]. Therefore, when preserving the LCA during rectal cancer surgery, preoperative planning of colonic mobilization maneuvers is essential to achieve both complete tension-free anastomosis and adequate proximal resection margin, ultimately optimizing oncologic outcomes.

Few studies have examined lengthening procedures in cadavers, which provide a safe environment for meticulous scientific evaluation. In this study, laparoscopic surgical procedures were simulated in initially fixed cadaveric specimens. By performing the key steps of complete mobilization of the splenic flexure, high ligation of the IMV, and division of the IMA at its root, we aimed to measure the additional length gained in the resected specimen while preserving the LCA. This measurement was conducted while meeting two core clinical requirements: (1) obtaining an adequate proximal resection margin and (2) ensuring a completely tension-free anastomosis. With preservation of the LCA, the mean length of the colonic specimen resected to achieve a completely tension-free anastomosis at the pelvic floor (a “resting” anastomotic state [2]) was 10.4 ± 6.9 cm. Among these, the length of the resected colon was less than 10 cm in 10 cadaveric specimens. Therefore, under these conditions, extended mobilization is necessary to achieve an adequate proximal resection margin. After complete SFM in all cadaveric specimens, the colon gained an average length of 6.3 ± 1.7 cm. The total resection length exceeded 10 cm in all cases.

Thum-Umnuaysuk et al. quantified colon lengthening in 13 cadavers fixed with formalin using four mobilization techniques: (1) low IMA ligation; (2) high IMA ligation; (3) high IMA ligation + complete SFM; (4) high IMA ligation + complete SFM + high IMV ligation. Measurements from the colosigmoid junction to pubic symphysis revealed progressive lengthening: (1) 2.08 ± 4.39 cm → (2) 5.02 ± 5.51 cm → (3) 8.20 ± 5.95 cm → 4)17.98 ± 6.80 cm [15]. For comparison, in our study, complete SFM resulted in greater elongation of the colon—averaging 6.3 ± 1.7 cm. This discrepancy may stem from our use of fresh-frozen cadavers with only preliminary formalin fixation, contrasting with the fully formalin-fixed specimens in Thum-Umnuaysuk et al.‘s study. Crucially, literature documents a 15.25% physiologic shortening in freshly resected specimens ex vivo, escalating to 36.3% after 24-hour formalin fixation [16]. The colonic lengthening achieved by our SFM technique (6.3 ± 1.7 cm) closely aligned with the 5.8 ± 3.7 cm reported in Reddy et al.‘s prospective study of 27 rectal cancer patients, further supporting the clinical validity of our cadaveric measurements [17]. Thus, our model more accurately simulates in vivo conditions.

A limitation of this study is that the baseline measurement (10.4 ± 6.9 cm) was obtained with an intact IMV, a condition that isolates the gain from subsequent high ligation but differs from the standard clinical practice, where the IMV is often divided earlier. Consequently, the reported baseline likely underestimates the bowel length routinely available during LCA-preserving surgery, and the proportion of cases requiring additional maneuvers in this experimental setting may be inflated relative to the actual surgical experience. Furthermore, the sequential experimental design of measuring SFM alone and then adding high IMV ligationdoes not fully replicate clinical practice, where these maneuvers are often performed together. The reported 14.4 cm gain from high IMV ligation represents the additional lengthening achieved after complete SFM. However, the independent effect of high IMV ligation alone was not directly assessed. Future studies employing a parallel-group design may provide data that more closely mirror the intraoperative decision-making.

**Fig. 3:**
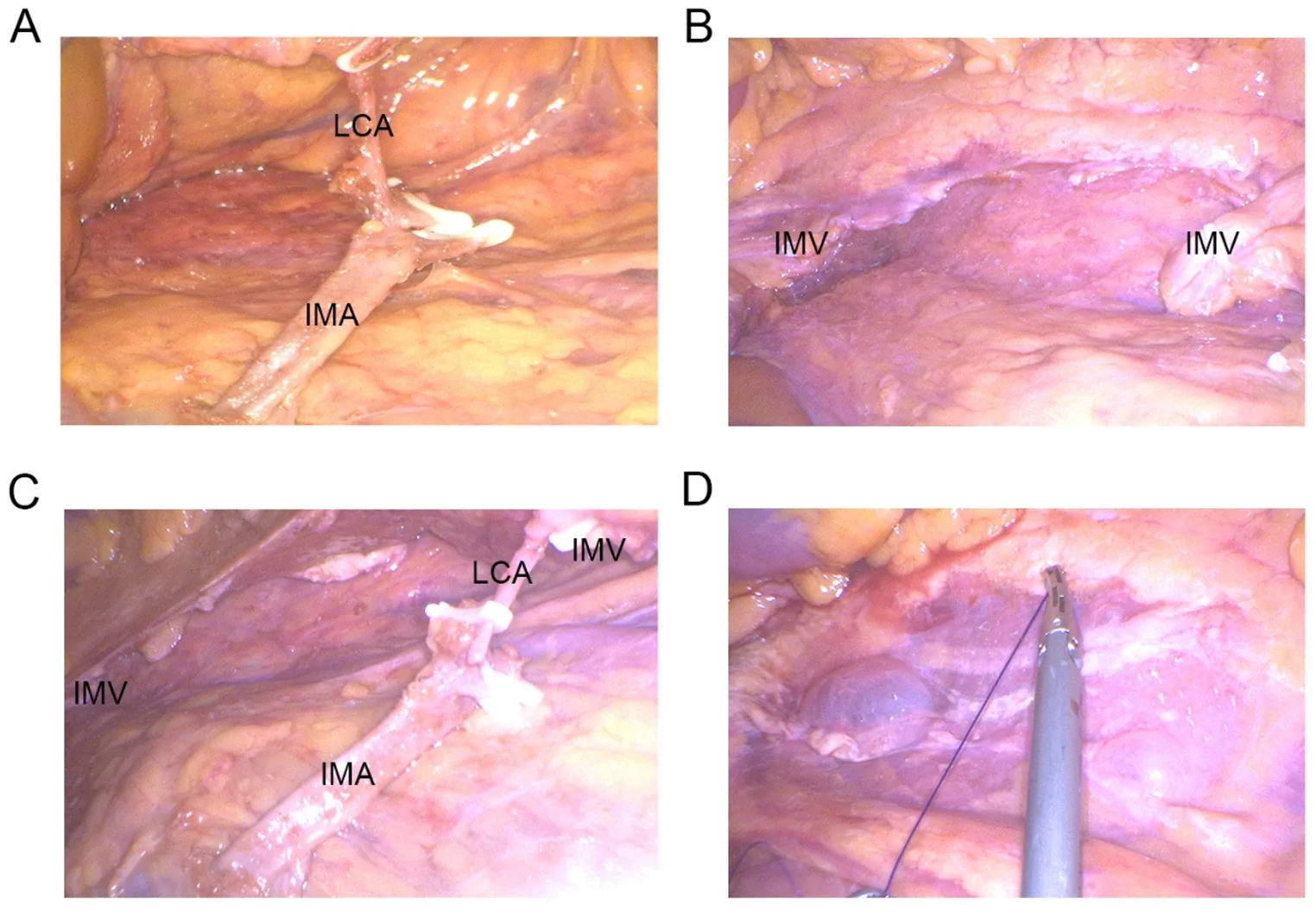
The maximum length gained was after high ligation of IMV. **A**. Preservation of the LCA, when the IMV has not been ligated at a high level, the course of the LCA opposes the direction needed to lengthen the bowel for tension-free anastomosis; **B**. High ligation of the IMV; **C**. After high ligation of the IMV, the direction of the LCA becomes downward, aligning with the bowel elongation pathway; **D**. The distance from the IMV to the marginal artery arcade

Similar to the findings reported by Thum-Umnuaysuk et al. [15], high ligation and transection of the IMV near the pancreas resulted in a mean intestinal lengthening of 14.4 ± 2.1 cm, the maximum elongation observed, attributable to mesenteric release. When the IMV was preserved, the LCA maintained its cephalad orientation, restricting the caudal elongation of the bowel due to countertraction from the mesentery (Fig. 3A). After high ligation and transection of the IMV, mesenteric traction was eliminated, allowing intestinal lengthening as the LCA was repositioned caudally (Fig. 3B and C). The degree of intestinal lengthening correlated directly with the distance between the IMV and the marginal arterial arch. A greater distance permitted more extensive mesenteric release after IMV transection, resulting in greater bowel elongation. However, in patients with persistent descending mesocolon (PDM), however, the IMV is located in close proximity to the marginal arterial arch, a characteristic vascular anatomical feature of this congenital anomaly [18]. This proximity necessitates cautious surgical planning: excessively high transection should be avoided to prevent marginal arch injury, and mesenteric release is relatively limited, achieving only modest lengthening of the bowel.

In this study, we simulated the surgical procedure of laparoscopic anterior resection for rectal cancer using frozen cadavers. Through three bowel-lengthening techniques, we quantified the additional bowel length gained by performing complete SFM, high ligation of the IMV, and high ligation of the IMA while preserving the LCA. This was done to achieve both tension-free colorectal anastomosis in the pelvic cavity and adequate proximal resection margins. For surgeries requiring resection of two bowel segments in patients with multiple primary colon cancers, or extended proximal bowel resection due to radiation enteritis, we recommend intraoperative preservation of the LCA. However, this typically necessitates complete SFM and/or high ligation of the IMV to achieve both tension-free anastomosis and adequate proximal resection margins. Complete SFM primarily gains bowel length, while high ligation of the IMV significantly reduces mesenteric tension. Our data demonstrate that high IMV ligation provides the greatest elongation of the mesenteric bowel segment. However, extreme caution is warranted when performing high IMV ligation in patients with malrotation of the descending colon.

Preoperative planning should prioritize high IMV ligation for optimal lengthening when preserving the LCA, particularly in patients with standard anatomy. SFM remains essential for baseline lengthening (mean 6.3 cm), whereas high IMA ligation (mean 18.4 cm) may be reserved for cases requiring extensive proximal resection, where LCA preservation is not the objective. Additionally, surgeons should preoperatively assess left colonic rotation (e.g., via CT angiography) to anticipate reduced IMV efficacy in malrotation and adapt techniques accordingly. These quantitative data provide a roadmap for achieving radical oncologic resection while minimizing anastomotic complications in LCA-preserving rectal cancer surgery.

This study has several limitations: [1] as a fresh cadaveric study, tissue elasticity and perfusion differ from in vivo conditions; [2] the sample size is small (*n* = 18); [3] the specimen population is predominantly male (14/18); and [4] despite standardized landmarks, some observer dependency in measurement cannot be fully excluded.

## Conclusion

In this fresh cadaveric model simulating laparoscopic low rectal cancer surgery with LCA preservation:

1. High ligation of the IMV emerged as the most effective applicable lengthening maneuver, providing a mean gain of 14.4 ± 2.1 cm while preserving LCA. This step eliminates mesenteric traction and allows caudal repositioning of the LCA, maximizing bowel elongation without compromising vascularity.
2. Complete SFM contributed an additional 6.3 ± 1.7 cm and served as an essential preliminary step for lengthening.
3. Anatomical variants critically impact outcomes: in descending colon malrotation, the proximity of the IMV to the marginal arterial arch reduces IMV ligation efficacy and necessitates caution to avoid vascular injury.
